# Meconium Microbiome Maturation Patterns Linked to Postnatal Growth Failure in Neonates

**DOI:** 10.3390/nu18132051

**Published:** 2026-06-23

**Authors:** Hyun Ho Kim, Sae Yun Kim, Hyun-Mi Kang, Young-Ah Youn

**Affiliations:** Department of Pediatrics, Seoul St. Mary’s Hospital, College of Medicine, The Catholic University of Korea, 222 Banpo-daero, Seocho-gu, Seoul 06591, Republic of Korea; gushkrs@gmail.com (H.H.K.); pedhmk@catholic.ac.kr (H.-M.K.); lea732@hanmail.net (Y.-A.Y.)

**Keywords:** meconium microbiome, gut microbiota, postnatal growth failure, preterm infants, microbial maturation

## Abstract

**Background/Objectives**: This study investigated whether meconium microbial profiles differed according to postnatal growth failure (PGF) and whether gestational age (GA)-related microbial maturation patterns appeared to vary according to subsequent growth status. **Methods**: Meconium samples were collected from 310 neonates born at 22–40 weeks of gestation, and 16S rRNA sequencing was conducted. After excluding small-for-gestational-age infants, the analyses included 151 samples from PGF+ infants and 131 samples from PGF− infants. Microbial composition, alpha and beta diversity were compared according to PGF status. Distance-based redundancy analyses (dbRDA) were conducted to evaluate the independent association between PGF and microbiome composition. **Results**: The core meconium microbiome differed between groups. PGF+ infants showed a predominance of Proteobacteria (36.60% vs. 27.96%), whereas PGF− infants had relatively higher abundances of Firmicutes (35.47% vs. 30.64%) and Bacteroidetes (28.96% vs. 26.19%) than PGF+ infants. GA-related microbial maturation patterns also differed between groups. At the genus level, the PGF− group showed significant positive correlations with GA for *Faecalibacterium*, *Sutterella*, *Dialister*, *Megamonas*, *Escherichia*/*Shigella*, and *Roseburia* after false discovery rate correction, whereas no genus-level correlation remained significant in the PGF+ group. Alpha diversity did not differ significantly between groups, whereas beta diversity differed modestly (*R*^2^ = 0.0087, *p* = 0.042). After adjustment for GA and birth weight, the PGF effect remained significant in the Bray–Curtis-based dbRDA model, whereas it was not significant in the Jaccard-based model. **Conclusions**: PGF was associated with abundance-based shifts within shared meconium taxa, suggesting subtle differences in early microbial developmental patterns among infants who later developed PGF.

## 1. Introduction

The human gut microbiota contributes to early-life health by shaping immune maturation, regulating host defense against opportunistic pathogens, and modulating intestinal endocrine and metabolic functions [1,2,3]. Accordingly, perturbations in early microbial assembly have been associated with adverse outcomes, including infection, intestinal inflammation, impaired nutrient use, and altered neurodevelopmental trajectories [4,5,6,7,8].

Despite substantial improvements in neonatal intensive care, very preterm infants remain at high risk of mortality, major morbidities, and adverse long-term neurodevelopmental outcomes [9,10]. Postnatal growth failure (PGF) is one of the most common problems in preterm infants and has been associated with higher mortality and poorer neurodevelopmental outcomes [11,12]. Optimal postnatal growth is a key determinant of later growth and neurodevelopment [13,14]. In preterm infants, growth is influenced by nutritional intake, intestinal maturation, inflammation, delayed enteral feeding, antibiotic exposure, and the overall severity of illness [15,16]. Many of these same factors shape early microbial colonization, including maternal microbial transfer during birth and through breast milk [17]. The early gut microbiome may contribute to postnatal growth through interconnected pathways involving short-chain fatty acid (SCFA)–mediated energy metabolism, nutrient absorption, and intestinal immune development, suggesting a potential microbiota–immunity–growth axis during neonatal development [18,19,20]. Alterations in this early microbial environment may therefore reflect or contribute to growth vulnerability during a critical developmental window [21,22]. Thus, the gut microbiome may serve as a key biological interface linking prematurity-related exposures to subsequent growth outcomes.

However, despite growing interest in the neonatal microbiome, most previous studies have focused broadly on prematurity, low birth weight, necrotizing enterocolitis, or sepsis rather than on postnatal growth patterns or nutrition-related growth vulnerability [23,24,25]. Whether meconium microbiome profiles and gestational age (GA)-related microbial maturation patterns differ by postnatal growth status remains unclear. This distinction is important because GA strongly influences microbial maturation [26], whereas nutritional support after birth may mediate or modify the relationship between early microbial composition and later growth [21,22]. Therefore, characterizing the meconium microbiome in relation to postnatal growth may provide insight into the early microbiome–nutrition–growth axis in preterm infants.

In the present study, we collected first-pass meconium samples from a large cohort of Korean neonates with varying GA and analyzed microbial composition using 16S rRNA sequencing. We hypothesized that (i) the meconium microbiome composition would differ between neonates who subsequently developed postnatal growth failure (PGF+) and those who did not (PGF−), and (ii) GA-related microbial maturation may be less evident in the PGF+ group and associated with subsequent PGF. Based on this hypothesis, we investigated whether meconium microbial profiles differ by postnatal growth status, and whether growth status modified GA-related microbial maturation patterns. By integrating early microbial composition with subsequent growth outcomes, we aimed to explore whether the meconium gut microbiome might be associated with postnatal growth vulnerability among preterm infants in the context of nutritional support.

## 2. Materials and Methods

### 2.1. Study Population

This was a prospective cohort study conducted at the Catholic University of Korea, Seoul St. Mary’s Hospital, a tertiary referral university hospital with a level IV neonatal intensive care unit (NICU). The participants were neonates admitted to the NICU between May 2021 and September 2023. Neonates were included if they had a first meconium sample obtained within 72 h after birth during the study period, were admitted to the NICU regardless of GA at birth and had written informed consent from their legal guardians. Neonates were excluded if they were transferred to the NICU after initial admission to the nursery or another hospital, passed their first meconium stool at 72 h or more after birth, or were full-term infants primarily admitted for gastrointestinal problems. Additionally, infants born as small for gestational age (SGA), whose birth weight was < 10th percentile were excluded from the analyses.

Clinical data were collected from electronic medical records from admission to discharge. Baseline information, including maternal factors (age, obstetric complications, and mode of delivery), GA, birth weight, birth-related history, and major clinical outcomes, was collected.

### 2.2. Sample Collection and Fecal Microbiome Analysis Method

#### 2.2.1. Sample Collection

Stool samples were collected by NICU nurses as part of routine clinical care. All meconium samples were obtained within 72 h of birth. Immediately after passage, 1–2 mL of the first passed meconium was collected from the diapers using sterile spatulas, placed in sterile tubes, and stored at −20 °C. Samples were delivered to the laboratory (AIBIOTICS Co., Ltd., Changwon, Republic of Korea) within 14 days of collection and subsequently stored at −80 °C until DNA extraction for microbiome analysis.

#### 2.2.2. 16S rRNA Gene Sequencing and Microbiome Data Processing

DNA was extracted from meconium samples, and V3–V4 region of the 16S rRNA gene was amplified by PCR. Subsequently, indexing PCR process was performed to attach dual indices and Illumina sequencing using PCRBIO VeriFi Mix (PCR Biosystems^®^, London, UK) and Nextera^®^ Index kit V2 Set A (Illumina^®^, San Diego, CA, USA). After indexing, the final pooled library was assessed for concentration and fragment size. Sequencing data targeting bacterial V3–V4 hypervariable regions were processed using the 16S Metagenomics app and Quantitative Insights Into Microbial Ecology 2 (QIIME 2; versions 2020.2 and 2020.6) [27]. DNA reads less than 200 bp in length were excluded from the taxonomic analysis. The remaining reads were demultiplexed and denoised using DADA2 [28]. Denoised reads were trimmed at 15 bp and truncated at 260 bp, and chimeric reads were removed. The R package decontam (version 1.8.0) was used to identify and remove environmental contaminants from each sample type [29]. Detailed methods for meconium sample collection, preprocessing, and analysis are provided in Appendix A.1.

### 2.3. Definitions

Postnatal growth failure (PGF) was defined as a discharge weight below the 10th percentile for postmenstrual age according to the Fenton growth chart [30]. Infants were therefore classified into the PGF+ group (discharge weight < 10th percentile) and the PGF− group (discharge weight ≥ 10th percentile).

Antenatal corticosteroid (ACS) was defined as the administration of at least one antenatal corticosteroid dose to the mother before delivery for fetal lung maturation. Prolonged premature rupture of membranes was defined as rupture of membranes lasting > 18 h before delivery. Histological chorioamnionitis was defined as described by Yoon et al. [31]. GA at birth was calculated based on the last menstrual period or first-trimester ultrasonography. SGA was defined by birth weight below the 10th percentile for GA and sex [30]. Sepsis was defined as culture-proven sepsis based on positive blood culture results. Necrotizing enterocolitis (NEC) was defined as stage II or higher [32]. Feeding intolerance (FI) was defined as persistent gastric residuals exceeding 50% of the previous feeding volume, with or without increased abdominal girth, in the absence of culture-proven sepsis or radiographic evidence of NEC for 48 h [33], or recurrent gastric residuals more than three times per day that prevented feeding advancement by >10–20 mL/kg/day. Time to full feeding was defined as the number of days required to achieve enteral feeding of >100 mL/kg/day.

### 2.4. Statistical Analyses

Clinical characteristics and microbiological data were compared between infants in the PGF+ and PGF− groups. Continuous variables are described mean ± standard deviation or median (interquartile range [IQR]), and categorical variables are presented as frequencies and percentages, as appropriate. For group comparisons in continuous variables, we used the Mann–Whitney U test. Differences in categorical variables are compared using Fisher’s exact test; although all expected cell counts exceeded 5 and Pearson’s chi-square test was therefore applicable, Fisher’s exact test was used to obtain exact *p*-values. Alpha diversity was calculated using Shannon’s diversity index, and beta diversity was visualized by Principal Coordinate Analysis (PCoA) based on Bray–Curtis and Jaccard distance/dissimilarity matrices Distance-based redundancy analysis (dbRDA) was performed to evaluate whether PGF status was associated with meconium microbiome composition after adjustment for GA and birth weight [34]. Spearman’s rank correlation analysis was used to evaluate associations between GA and relative abundance of selected taxa within each PGF group using Benjamini–Hochberg false discovery rate correction. All statistical tests were 2-sided, and between-group differences or correlations coefficient were considered significant at *p* < 0.05. All statistical analyses were performed using SPSS, version 24 software (IBM Corp., Armonk, NY, USA) and R software package version 4.3.0 (R Foundation for Statistical Computing, Vienna, Austria).

### 2.5. Ethical Statement

The study protocol was reviewed and approved by the Human Research Ethics Board of Seoul St. Mary’s Hospital (IRB No. KC22RISI0240, approved on 14 April 2022, and KC22TNSI0297, approved on 3 June 2022). Stool samples and clinical data were prospectively collected after obtaining written informed consent from the infant’s legal guardians upon admission.

## 3. Results

### 3.1. Study Cohort, Demographic, and Clinical Information

During the study period, first-pass meconium samples were prospectively collected from 386 preterm and full-term infants born at 22 to 40 weeks of gestation and admitted to the NICU of Seoul St. Mary’s Hospital. After preprocessing, 76 samples were excluded because they were unsuitable for analysis. Thus, 310 infants were initially included in the cohort. After the exclusion of SGA infants, 282 infants remained, of whom 151 were classified as PGF+ and 131 as PGF−.

Compared with the PGF− group, the PGF+ group was more frequently associated with assisted pregnancy by IVF (23.8% vs. 11.5%, *p* = 0.008), ACS exposure (64.9% vs. 37.4%, *p* < 0.001), preeclampsia (19.9% vs. 8.4%, *p* = 0.007), and caesarean section delivery (93.4% vs. 82.4%, *p* = 0.005). In contrast, histologic chorioamnionitis was less frequent in the PGF+ group than in the PGF− group (0.7% vs. 6.9%, *p* = 0.007). GA and birth weight were significantly lower in the PGF+ group than in the PGF− group (34 [33–36] vs. 37 [31–38] weeks, *p* = 0.001; 2210 [1918–2544] vs. 3000 [1810–3295] g, *p* < 0.001, respectively) (Table 1). Regarding neonatal outcomes, both groups did not differ significantly in admission hypothermia, respiratory distress syndrome, intraventricular hemorrhage, periventricular leukomalacia, parenteral nutrition (PN) duration, time to full feeding, length of hospital stay, or mortality. However, the PGF− group had significantly higher rates of symptomatic patent ductus arteriosus (9.2% vs. 1.3%, *p* = 0.004), NEC or FI (20.6% vs. 8.6%, *p* = 0.006), sepsis (9.9% vs. 2.0%, *p* = 0.004), and retinopathy of prematurity requiring treatment (5.3% vs. 0.0%, *p* = 0.004) than the PGF+ group (Table 2).

In the subgroup analysis of infants over 28 weeks of gestation, infants in the PGF+ group had significantly lower GA and birth weight than those in the PGF− group (34.0 [33.0–36.0] vs. 37.0 [34.0–38.0] weeks, *p* < 0.001; 2215.5 [1933.3–2547.0] vs. 3106.0 [2647.0–3311.3] g, *p* < 0.001, respectively). Admission hypothermia was more frequent in the PGF+ group than in the PGF− group (79.3% vs. 67.0%, *p* = 0.032). In addition, the PGF+ group had a longer duration of PN than the PGF− group (4.0 [3.0–6.0] vs. 3.0 [0.75–5.25] days, *p* = 0.027). Although respiratory distress syndrome, intraventricular hemorrhage, periventricular leukomalacia, and mortality were more common in the PGF+ group than in the PGF− group, the differences were not statistically significant. Length of stay also tended to be longer in the PGF− group; the difference was not statistically significant (Table A1).

### 3.2. Comparison of Microbiomes According to Postnatal Growth

**Figure 1:**
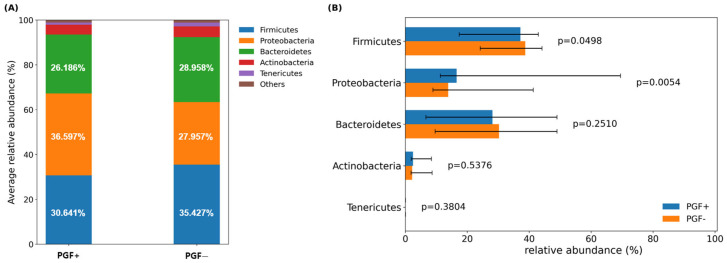
Gut microbiome comparison between two groups at the phylum level. (**A**) Stacked bar showing plots the average relative abundance of top 5 phyla in the PGF+ and PGF− groups, (**B**) Horizontal bar graph showing the median relative abundance of the top 5 phyla in the PGF+ and PGF− groups, with error bars indicating the interquartile range. *p* values were calculated using the Mann–Whitney U test. Abbreviations: IQR, interquartile range; PGF, postnatal growth failure.

At the phylum level, the core microbiome was Firmicutes (32.9%), followed by Proteobacteria (32.6%), and Bacteroidetes (27.5%). Compared with the PGF+ group, the PGF− group showed a significantly higher relative abundance of Firmicutes (35.427% vs. 30.641%, *p* = 0.0498), whereas Proteobacteria was significantly lower in the PGF− group (27.957% vs. 36.597%, *p* = 0.005). The relative abundances of Bacteroidetes (28.958% vs. 26.186%, *p* = 0.251), Actinobacteria (4.781% vs. 4.404%, *p* = 0.538), Tenericutes (1.618% vs. 0.995%, *p* = 0.380), and others (1.259% vs. 1.178%, *p* = 0.970) did not differ significantly between the two groups (Figure 1 and Table A2).

**Figure 2:**
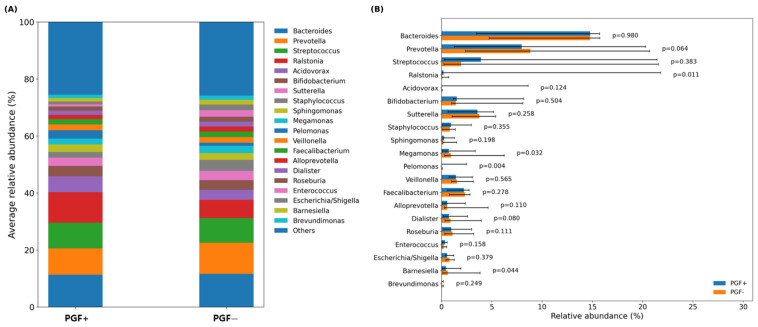
Gut microbiome comparison between two groups at the genus level. Stacked bar plot showing the average relative abundance of the top 20 genera in the PGF+ and PGF− groups (**A**), Horizontal bar graph showing the median relative abundance of the top 20 genera in the PGF+ and PGF− groups, with error bars indicating the interquartile range (IQR) (**B**). *p* values were calculated using the Mann–Whitney U test. Abbreviations: PGF, postnatal growth failure.

The most abundant genera were *Bacteroides* (11.5%), *Prevotella* (10.0%), *Streptococcus* (8.9%), and *Ralstonia* (8.7%). Among the top 20 genera, *Ralstonia* was more abundant in the PGF+ group than in the PGF− group (10.688% vs. 6.350%, *p* = 0.011), whereas *Megamonas* (2.074% vs. 2.502%, *p* = 0.032) and *Barnesiella* (1.231% vs. 1.609%, *p* = 0.044) were lower in the PGF+ group. *Pelomonas* was higher in the PGF+ group than in the PGF− group (2.906% vs. 1.074%, *p* = 0.0037). *Prevotella* showed a borderline difference, with a higher relative abundance in the PGF− group (10.932% vs. 9.219%, *p* = 0.064). The relative abundances of the remaining genera did not differ significantly between the two groups. The proportion of others was similar between groups (25.514% vs. 25.769%, *p* = 0.967) (Figure 2 and Table A3).

### 3.3. Gestational Age and Microbiota Acquisition

**Figure 3:**
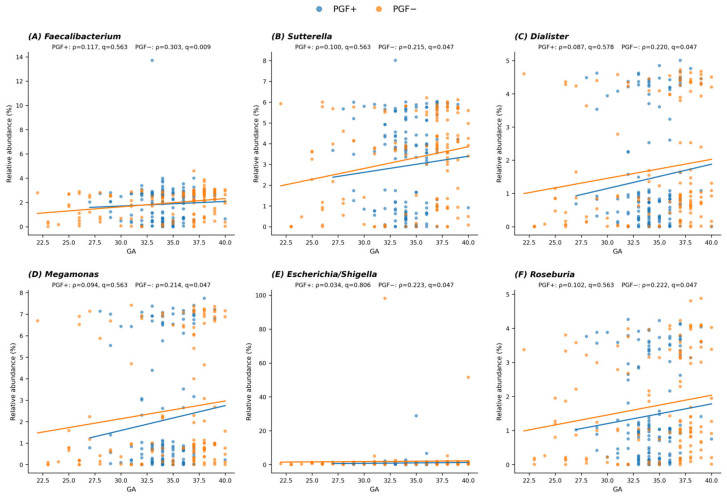
Relative abundance of selected genera according to GA. Scatter plots show the correlations between gestational age and the relative abundance of genera that remained significant after Benjamini–Hochberg FDR correction in the PGF− group. Linear trend lines are shown for visualization. Spearman’s correlation coefficients (ρ) and FDR-adjusted q values are displayed in each panel. The analyzed taxa were (**A**) *Faecalibacterium*, (**B**) *Sutterella*, (**C**) *Dialister*, (**D**) *Megamonas*, (**E**) *Escherichia*/*Shigella*, (**F**) *Roseburia*. Abbreviations: GA, gestational age; PGF, postnatal growth failure.

Spearman correlation analyses were performed to evaluate associations between GA at birth and the relative abundance of selected phylum- and genus-level taxa within each PGF group. *p*-values were adjusted for multiple comparisons using the Benjamini–Hochberg false discovery rate method separately for each group and taxonomic level. At the phylum level, no correlations remained statistically significant after FDR correction in either group. At the genus level, no taxa remained significant after FDR correction in the PGF+ group. In the PGF− group, several genera remained significantly positively correlated with GA after FDR correction: *Faecalibacterium* (Spearman’s ρ = 0.303, 95% CI: 0.139–0.452, q = 0.0085), *Sutterella* (ρ = 0.215, 95% CI: 0.045–0.373, q = 0.0474), *Dialister* (ρ = 0.220, 95% CI: 0.051–0.378, q = 0.0474), *Megamonas* (ρ = 0.214, 95% CI: 0.044–0.372, q = 0.0474), *Escherichia*/*Shigella* (ρ = 0.223, 95% CI: 0.054–0.380, q = 0.0474), and *Roseburia* (ρ = 0.222, 95% CI: 0.052–0.379, q = 0.0474) (Figure 3 and Table A4).

### 3.4. Microbial Diversity

**Figure 4:**
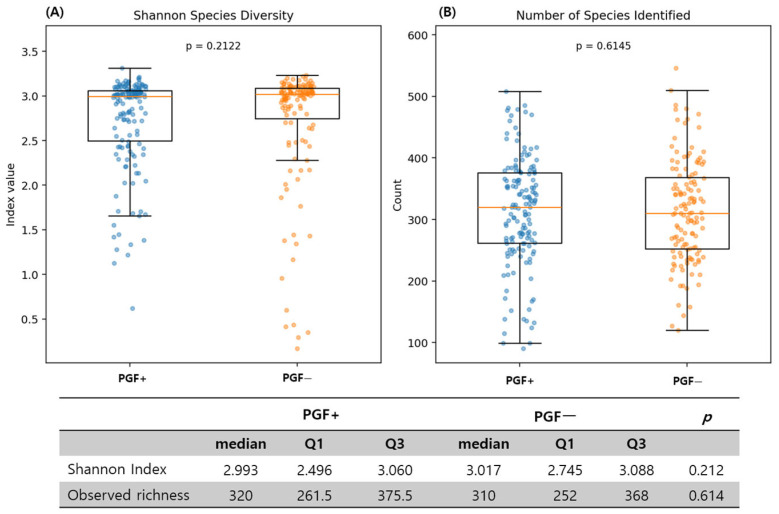
Genus-level alpha diversity according to PGF status. Boxplots with overlaid individual data points show genus-level alpha diversity in the PGF+ group and PGF− group. (**A**) Shannon index was described by Shannon species diversity. (**B**) Observed richness evaluated by number of species identified. The center line indicates the median, the box indicates the interquartile range, and whiskers indicate the data range excluding outliers. Abbreviations: PGF, postnatal growth failure.

Genus-level alpha diversity, assessed by the Shannon index and observed richness, did not differ significantly according to PGF status (Shannon index: median 2.993 vs. 3.017, *p* = 0.212; Number of Species Identified: median 320 vs. 310 genera, *p* = 0.614, PGF+ and PGF−, respectively). These findings suggest that overall genus-level microbial diversity and richness were comparable between infants with and without PGF (Figure 4).

**Figure 5:**
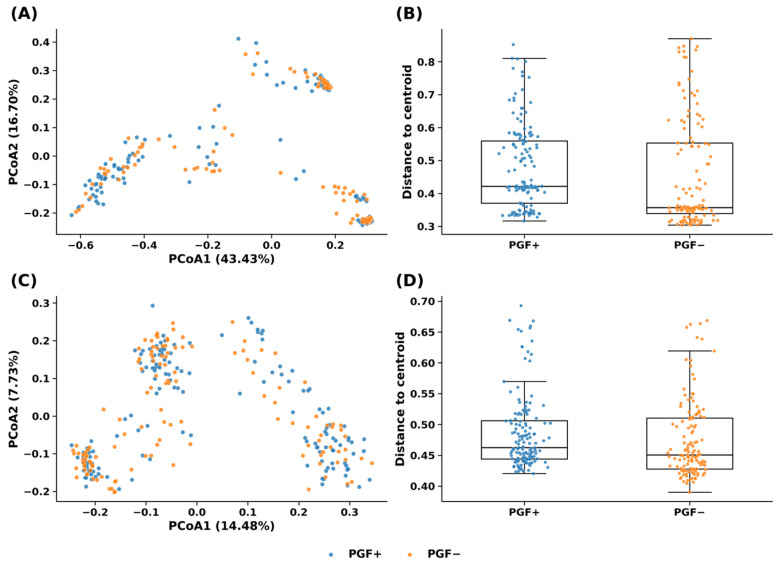
Comparison of beta diversities. (**A**) Bray–Curtis PCoA plot showing a statistically significant but very small difference in community structure between the PGF+ and PGF− groups (PERMANOVA *R*^2^ = 0.0087, *p* = 0.042). (**B**) Bray–Curtis beta-dispersion analysis showing no significant difference in within-group dispersion (PERMDISP *p* = 0.361). (**C**) Jaccard PCoA plot showing no significant difference in presence–absence-based microbial community membership between groups (PERMANOVA *R^2^* = 0.0046, *p* = 0.122). (**D**) Jaccard beta-dispersion analysis also showing no significant between-group difference in dispersion (PERMDISP *p* = 0.139). PGF+ infants are shown in blue, and PGF− infants are shown in orange. Abbreviations: PCoA, Principal Coordinates Analysis; PERMANOVA, Permutational Multivariate Analysis of Variance; PERMDISP, Permutational test for multivariate dispersion; PGF, postnatal growth failure.

Beta diversity was evaluated using Bray–Curtis and Jaccard distance and visualized by principal coordinate analysis (PCoA). Based on Bray–Curtis dissimilarity, a small but statistically significant difference in overall microbial community structure was observed between two groups (PERMANOVA: *R*^2^ = 0.0087, *p* = 0.042; Figure 5A). In contrast, Jaccard PERMANOVA showed no significant difference in presence–absence-based microbial membership (PERMANOVA: *R*^2^ = 0.0046, *p* = 0.122). Additionally, both beta dispersions did not reach statistical significance (Bray–Curtis: *F* = 1.483, *p* = 0.361; Jaccard: *F* = 2.524, *p* = 0.139). These findings suggest that PGF-associated differences in the meconium microbiome were more related to differences in the relative abundance distribution of shared taxa than to the presence or absence of distinct microbial members (Figure 5).

**Figure 6:**
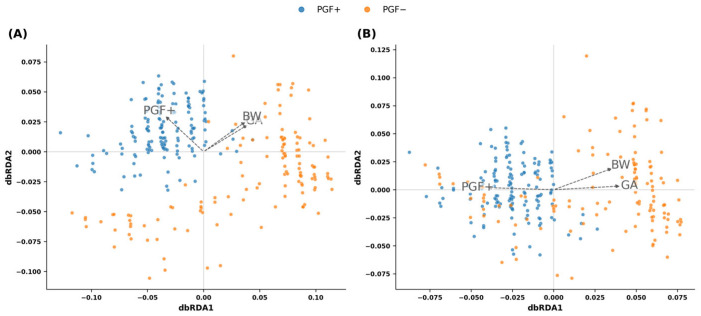
dbRDA biplots of the meconium microbiome and adjusting for GA and BW (**A**) Bray–Curtis-based dbRDA biplot. (**B**) Jaccard-based dbRDA biplot. Points represent individual samples, and colors indicate PGF group status. Arrows indicate the direction of association of the explanatory variables included in the dbRDA model. In the Bray–Curtis-based model, the overall model was significant (*F* = 2.271, *R*^2^ = 0.0239, *p* = 0.014), and the adjusted effect of PGF was also significant (*F* = 2.367, *R*^2^ = 0.0083, *p* = 0.047). In the Jaccard-based model, the overall model was significant (*F* = 1.257, *R*^2^ = 0.0134, *p* = 0.047), whereas the adjusted effect of PGF was not significant (*F* = 1.218, *R*^2^ = 0.0043, *p* = 0.156). For visualization purposes, a small amount of jitter was added to reduce overplotting; this did not affect the underlying dbRDA results. Abbreviations: BW, birth weight; dbRDA, Distance-based redundancy analysis; GA, gestational age; PGF postnatal growth failure.

dbRDA was performed to assess whether PGF status was associated with meconium microbiome composition after adjustment for GA and birth weight. In the Bray–Curtis-based dbRDA model, the overall model was statistically significant (*F* = 2.271, *R*^2^ = 0.0239, *p* = 0.014), and the adjusted effect of PGF also remained significant after adjustment for GA and birth weight (*F* = 2.367, *R*^2^ = 0.0083, *p* = 0.047). In contrast, in the Jaccard-based dbRDA model, although the overall model was significant (*F* = 1.257, *R*^2^ = 0.0134, *p* = 0.047), the adjusted effect of PGF was not significant (*F* = 1.218, *R*^2^ = 0.0043, *p* = 0.156). These findings suggest that, after adjustment for GA and birth weight, PGF status was independently associated with abundance-based differences in meconium microbial community composition, but not with presence–absence-based differences in microbial community membership (Figure 6).

## 4. Discussion

The neonatal microbiota undergoes rapid diversification after birth [26]. Our study revealed that growth status at discharge was associated with differences in meconium gut microbial composition, offering valuable insights into the impact of the immediate postnatal growth status on gut dysbiosis in newborns. First, at the phylum level, infants without PGF showed a higher relative abundance of Firmicutes and a lower relative abundance of Proteobacteria. At the genus level, several taxa, including *Megamonas* and *Barnesiella* were enriched in the PGF− group, whereas *Ralstonia* and *Pelomonas* were relatively more abundant in the meconium of PGF+ group. This dysbiotic pattern parallels the Enterobacteriaceae-dominated microbial succession described in hospitalized preterm infants [35]. Overall, these findings suggest that early gut microbiome development is closely associated with postnatal growth status in newborns. Recent data suggest that perinatal factors influence meconium microbiota composition more strongly than prenatal factors [36]. Second, through stratification by postnatal growth at discharge, GA-related microbial maturation patterns differed. Increasing GA was associated with higher relative abundance of several genera, including *Faecalibacterium*. These positive associations were generally stronger and more consistent in the PGF− group. This pattern aligns with longitudinal evidence of preterm gut microbiota maturation in favorable clinical trajectories [37]. Third, although the alpha diversity and the number of identified species did not differ significantly between the PGF+ and PGF− groups, beta diversity based on Bray–Curtis dissimilarity differed significantly between groups. These findings suggest that the meconium microbiome may serve as a potential early indicator of growth vulnerability. If infants at higher risk can be identified early, individualized nutritional support and closer monitoring of enteral feeding progression may help prevent or mitigate PGF.

In the meconium of infants in the PGF− group, we observed a higher relative abundance of Firmicutes and a lower relative abundance of Proteobacteria. Firmicutes include several taxa involved in energy harvest [26] and SCFA production, particularly butyrate-producing anaerobes [38]. Because SCFAs, especially butyrate, serve as important energy sources for intestinal epithelial cells and support gut barrier function, enrichment of SCFA-producing taxa may reflect more advanced microbial maturation and favorable intestinal energy homeostasis [39]. Consistent with this concept, An et al. reported that SGA rats with catch-up growth showed altered gut microbial composition and increased fecal SCFA levels, suggesting a role for SCFAs in postnatal weight gain [40]. Thus, the higher abundance of Firmicutes in the PGF− group may reflect a microbial profile more favorable for energy metabolism, intestinal maturation, and early postnatal growth, consistent with previous studies [41].

Conversely, the higher abundance of Proteobacteria in the PGF+ group may be relevant to subsequent neonatal growth trajectories. As we previously reported, Proteobacteria-dominant communities may represent a feature of microbial immaturity and dysbiosis in preterm infants [26,42]. The expansion of Proteobacteria is commonly considered a marker of microbial immaturity and dysbiosis [43]. Because many Proteobacteria are Gram-negative bacteria rich in lipopolysaccharide, their predominance has been linked to innate immune activation and intestinal inflammation, which may in turn relate to nutrient absorption, metabolic homeostasis, and postnatal growth [4]. For example, a high burden of Enterobacteriaceae, a family within the phylum Proteobacteria, may be associated with growth failure through inflammation mediated by toll-like receptor 4 activation and insulin-related metabolic pathways [4,44].

Consistent with these phylum-level findings, genus-level analysis suggested subtle PGF-related differences in microbial composition. However, although beta-diversity differed modestly according to PGF status by PERMANOVA (*R^2^* = 0.0087, *p* = 0.036), the very small effect size suggests limited separation of microbial communities between groups. The PGF− group showed enrichment of *Megamonas*, a Firmicutes genus, and *Barnesiella*, a gut-associated anaerobic taxon, whereas the Proteobacteria genera *Ralstonia* and *Pelomonas* were enriched in the PGF+ group. These differences suggest that infants without PGF may harbor a more gut-adapted, anaerobe-enriched, and metabolically favorable microbial profile, whereas infants with PGF may exhibit features of delayed microbial maturation and a less stable intestinal environment, with enrichment of taxa often associated with environmental sources or opportunistic colonization [45]. Collectively, our findings support previous evidence that PGF is associated with delayed microbial maturation, characterized by Enterobacteriaceae predominance and depletion of strictly anaerobic taxa, whereas favorable postnatal growth is linked to a more mature gut microbial profile [4,46]. Longitudinal studies have also shown that early postnatal microbiota development is closely related to subsequent growth trajectories, and that NICU-related exposures, including antibiotic treatment and prolonged parenteral nutrition, can perturb early microbial assembly [4,26,47].

Taken together, our findings suggest that infants with PGF may have an unfavorable early microbial profile characterized by reduced metabolic support, delayed microbial maturation, and greater inflammatory potential. In contrast, infants without PGF appear to harbor a more gut-adapted and metabolically favorable microbial profile. These findings highlight the potential value of the meconium microbiome for identifying infants who may benefit from closer nutritional monitoring and individualized nutritional support.

In the initial analysis, the PGF− group had more complicated hospital courses, a counterintuitive finding likely driven by GA imbalance, which may have confounded the comparison between the PGF− and PGF+ groups, as extremely preterm infants are more likely to experience prolonged hospitalization, respiratory support, prolonged PN, and major prematurity-related morbidities. Thus, the worse clinical profile initially observed in the PGF− group may have reflected lower GA rather than PGF status itself. After restricting the analysis to infants born at ≥28 weeks, infants in the PGF+ group showed more complicated hospital courses (Table A1), supporting the link between PGF, clinical vulnerability, and delayed microbial maturation.

The gut microbiota of preterm infants at birth is less diverse than that of full-term infants and is highly vulnerable to dysbiosis because of physiological immaturity and postnatal exposures that disrupt microbial succession [26]. In this study, GA-related microbial variation was more pronounced in the PGF− group than in the PGF+ group with several genera, including *Sutterella*, *Megamonas*, *Faecalibacterium*, *Dialister*, *Roseburia*, and *Escherichia*/*Shigella*, showing positive correlations with GA only among infants of PGF− group. Notably, several of these taxa, particularly *Faecalibacterium*, *Roseburia*, *Dialister*, and *Megamonas*, are anaerobic or carbohydrate fermenting genera associated with gut microbial maturation and SCFA-related metabolic functions [4,16,48,49]. The GA-related increase in *Faecalibacterium*, a health-associated anti-inflammatory commensal that becomes more prominent with postnatal maturation, further supports the interpretation that infants without PGF may exhibit a more coordinated GA-related microbial maturation pattern [50,51]. However, because taxa such as *Sutterella* and *Escherichia*/*Shigella* may reflect mucosal host–microbe interactions or early colonization rather than mature SCFA-producing communities, and because 16S rRNA sequencing does not directly measure microbial metabolic activity, these functional implications should be interpreted cautiously [4,16,48].

An interesting finding was that alpha diversity did not differ significantly between groups, whereas beta diversity did. This suggests that the distinction between PGF+ and PGF− may not lie in the overall richness or evenness of the microbiome, but rather in differences in the relative composition. The contrasting results of the Bray–Curtis and Jaccard analyses are biologically interpretable because the two beta-diversity metrics capture different dimensions of microbial community variation; Bray–Curtis reflects quantitative differences in relative abundance, whereas Jaccard reflects qualitative differences in presence–absence-based microbial community membership [52]. This pattern suggests that PGF-associated microbial differences primarily reflected quantitative shifts in the relative abundance of shared taxa within a broadly shared meconium microbial community, rather than major qualitative differences related to the acquisition or loss of distinct taxa [53].

The adjusted dbRDA analysis further supports the interpretation that PGF-associated microbiome differences primarily reflected quantitative shifts in relative abundance. Even after adjustment for GA and birth weight, the PGF effect remained significant in the Bray–Curtis-based model, whereas it was not significant in the Jaccard-based model. This pattern suggests that PGF may be associated with redistribution of relative abundances among broadly shared taxa, rather than the acquisition or loss of distinct microbial members.

Adequate nutrition may serve as an important clinical pathway linking the early gut microbiome to postnatal growth in preterm infants [4,7]. In our cohort, infants with PGF required longer PN duration and achieved full enteral feeding later than those without PGF, suggesting that delayed enteral nutritional progression may be associated with both impaired growth and altered microbial development. Because enteral feeding provides substrates that promote microbial succession and the expansion of gut-adapted anaerobic taxa, prolonged PN and delayed full enteral feeding may reflect a delayed transition toward a microbial environment favorable for intestinal maturation and growth [54,55,56]. Importantly, the meconium microbiome may provide early insight into growth vulnerability; in particular, a Proteobacteria-dominant profile may reflect delayed or disrupted microbial maturation, supporting the need for closer nutritional surveillance and individualized nutritional support to promote timely enteral feeding advancement and adequate postnatal growth [4,7].

The strengths of our study include its prospective design and, to our knowledge, the largest Korean neonatal cohort to date examining the association between the meconium microbiome and postnatal growth. This cohort enabled us to explore growth-related microbial patterns across a broad range of GAs and provides insight into early gut dysbiosis in neonates with poor postnatal growth.

However, several limitations should be acknowledged. First, although adjusted Bray–Curtis dbRDA demonstrated significant differences in microbial community structure between the PGF+ and PGF− groups, the findings should be interpreted cautiously. Residual confounding by GA-related developmental maturity, unmeasured maternal or perinatal factors, and unavailable nutritional data cannot be completely excluded. In addition, differences in beta-dispersion between groups suggest that beta-diversity results may partly reflect differences in within-group variability rather than only differences in community composition. Second, because of the observational design, the observed microbiome differences should be interpreted as associations rather than evidence of a causal relationship with postnatal growth failure. Third, several methodological limitations should be acknowledged. Batch-related technical variation cannot be completely excluded because DNA extraction and amplification were performed at different time points. Furthermore, although 16S rRNA gene sequencing is useful for profiling microbial community composition, it has inherent limitations, including limited resolution for species- or strain-level classification and the inability to distinguish viable bacteria from residual DNA derived from nonviable bacteria. This approach also did not allow direct assessment of microbial functional pathways, and metabolomic, inflammatory biomarker, or other microbial functional analyses were not performed. Retrospective shotgun metagenomic sequencing was not feasible because of limited sample volume and the low-biomass nature of meconium. Fourth, because meconium is a low-biomass specimen, potential contamination from reagent, environmental, or laboratory sources cannot be completely excluded, particularly in genus-level analyses. Finally, the single-center design of this study may limit the generalizability of our findings. Future studies should validate these results in multicenter cohorts with greater demographic and ethnic diversity.

Future studies should validate these findings in multicenter cohorts with greater demographic and ethnic diversity. Longitudinal studies integrating serial microbiome sampling, detailed nutritional assessments, growth trajectories, and neurodevelopmental outcomes are needed to clarify the temporal and potential causal relationships between early microbial colonization and postnatal growth. Beyond compositional 16S rRNA profiling, the integration of shotgun metagenomics, metabolomics, including short-chain fatty acid and bile acid profiling, and inflammatory mediator profiling may help define the functional pathways underlying the microbiota–nutrition–growth axis. In addition, predictive models combining microbiome signatures with clinical variables should be developed and validated to determine whether early microbial profiles can improve risk stratification for postnatal growth failure [21]. Ultimately, interventional studies evaluating probiotics, prebiotics [57], human milk-derived bioactives, and personalized nutritional strategies are needed to determine whether modulation of the early-life microbiome can improve growth and developmental outcomes.

## 5. Conclusions

In summary, the meconium microbiome differed between infants with and without PGF. The PGF+ group showed Proteobacteria predominance, whereas the PGF− group had relatively higher abundances of Firmicutes and Bacteroidetes, suggesting a more mature and metabolically favorable microbial profile. GA-related microbial maturation patterns were also more evident in the PGF− group, with positive correlations between GA and several maturation-associated taxa. Although alpha diversity did not differ significantly between groups, beta-diversity analysis showed a statistically significant but small difference, suggesting subtle PGF-related shifts in overall microbial composition rather than a clearly distinct microbial profile. These findings suggest that the meconium microbiome may be closely linked to postnatal growth and optimal nutritional support and may help identify infants who could benefit from closer nutritional monitoring and individualized nutritional support in the context of PGF risk.

## Figures and Tables

**Table 1:** Perinatal characteristics of infants admitted to NICUs.

	PGF+ [*n* = 151]	PGF− [*n* = 131]	*p*
Assisted pregnancy, IVF	36 (23.8%)	15 (11.5%)	0.008
Antenatal corticosteroid	98 (64.9%)	49 (37.4%)	<0.001
PPROM > 18 h	17 (11.3%)	19 (14.5%)	0.476
Histologic chorioamnionitis	1 (0.7%)	9 (6.9%)	0.007
Maternal antibiotics use (>3 d)	130 (86.1%)	110 (84.0%)	0.620
Maternal DM	17 (11.3%)	20 (15.3%)	0.378
Oligohydramnios	6 (4.0%)	10 (7.6%)	0.206
Placenta abruption	4 (2.6%)	5 (3.8%)	0.738
Placenta previa	5 (3.3%)	8 (6.1%)	0.394
Preeclampsia	30 (19.9%)	11 (8.4%)	0.007
Caesarean section	141 (93.4%)	108 (82.4%)	0.005
Gestational age, weeks	34 [33,36]	37 [31,38]	0.001
Birth weight, g	2210.0 [1918.0, 2544.0]	3000.0 [1810.0, 3295.0]	<0.001
Male sex	90 (59.6%)	73 (55.7%)	0.547

Values are presented as means with median [IQR] for continuous variables or frequencies with percentages for categorical variables, as appropriate. *p*-values were calculated using Fisher’s exact test for categorical variables, and Mann–Whitney U test for continuous variables. Abbreviations: DM, diabetes mellitus; IVF, in vitro fertilization; IQR, interquartile range; NICU, neonatal intensive care unit; PGF, postnatal growth failure; PPROM, Prolonged premature rupture of membranes.

**Table 2:** Comparison of clinical outcomes during NICU admission.

	PGF+ [*n* = 151]	PGF− [*n* = 131]	*p*
Admission hypothermia (BT < 36.5)	120 (79.5%)	92 (70.2%)	0.097
Respiratory distress syndrome	46 (30.5%)	46 (35.1%)	0.446
symptomatic PDA	2 (1.3%)	12 (9.2%)	0.004
NEC or FI	13 (8.6%)	27 (20.6%)	0.006
Intraventricular hemorrhage	93 (61.6%)	75 (57.3%)	0.468
Periventricular leukomalacia	37 (24.5%)	41 (31.3%)	0.230
Sepsis	3 (2.0%)	13 (9.9%)	0.004
ROP requiring treatment	0 (0.0%)	7 (5.3%)	0.004
PN duration	4.0 [3.0, 6.0]	3.0 [1.0, 11.0]	0.949
Time to full feeding, d (>100 mL/kg/d)	5.0 [4.0, 6.0]	5.0 [4.0, 11.0]	0.326
Length of stay	16.0 [10.0, 25.5]	14.0 [9.0, 58.0]	0.651
Mortality	1 (0.7%)	0 (0.0%)	>0.999

Values are presented as means with median [IQR] for continuous variables or frequencies with percentages for categorical variables, as appropriate. *p*-values were calculated using Fisher’s exact test for categorical variables, and Mann–Whitney U test for continuous variables. Abbreviations: BT, body temperature; FI, feeding intolerance; NEC, necrotizing enterocolitis; NICU, neonatal intensive care unit; PDA, patent ductus arteriosus; PGF, postnatal growth failure; PN, parenteral nutrition; ROP, retinopathy of prematurity.

## Data Availability

The data presented in this study are available on request from the corresponding author due to privacy and ethical restrictions related to the use of confidential neonatal clinical information and institutional review board approval. The original contributions presented in this study are included in the article. Further inquiries can be directed to the corresponding author.

## Appendix A

### Appendix A.1. Details of Fecal Microbiome Analysis Method

#### Appendix A.1.1. DNA Extraction and Amplification

DNA was extracted from meconium samples using the Stool DNA/RNA Extraction Kit (TianLong Science and Technology Co., Xi’an, China), and DNA purity was assessed using a Nanodrop spectrophotometer. The V3–V4 hypervariable region of the bacterial 16S rRNA gene was amplified by target-specific PCR using VeriFi Mix Red (PCR Biosystems^®^, London, UK) and 16S-amplicon primer (Macrogen^®^, Seoul, Republic of Korea). Amplicon PCR was performed with an initial denaturation at 95 °C for 3 min, followed by 25 cycles of denaturation at 95 °C for 30 s, annealing at 55 °C for 30 s, and extension at 72 °C for 30 s, with a final elongation step at 72 °C for 5 min. The V3–V4 target region reverse primer (5′TCGTCGGCAGCGTCAGATGTGTATAAGAGACAGCCTACGGGNGGCW-GCAG3′) and forward primer (5′GTCTCGTGGGCTCGGAGATGTGTATAAGAGACAGGACT-ACHVGGGTATCTAATCC3′), according to the Illumina protocol, Preparing 16S Ribosomal RNA Gene Amplicons for the Illumina MiSeq System [58].

The amplified DNA subsequently were purified automatically in Nucleic Acid Extractor (TianLong Science and Technology Co.) using MagListo™ PCR/Gel Purification Kit (Bioneer^®^, Daejon, Republic of Korea), then the size and concentration were determined using Qsep100 (Bioptic^®^, Changzhou City, China). Purified samples were then standardized to 8 ng per reaction (total reaction volume of 20 µL) and Index PCR was performed to attach in dual indices and Illumina sequencing using PCRBIO VeriFi Mix (PCR Biosystems^®^, London, UK) and Nextera ^®^ Index kit V2 Set A (Illumina^®^, San Diego, CA, USA) at 95 °C for 3 min hotstart followed by 8 cycles of 95 °C for 30s and 55 °C for 30 s, 72 °C for 30 s with a final elongation step at 72 °C for 5 min. The indexed PCR products were purified, and DNA fragment size and concentration were assessed using a Qsep100 analyzer (Bioptic^®^, Changzhou City, China) and Qubit Flex Fluorometer (Invitrogen^®^, Thermo Fisher Scientific, Waltham, MA, USA), respectively.

#### Appendix A.1.2. Sequencing and Data Analysis

After DNA concentration and fragment size were assessed using Qubit and Qsep, respectively, the required pooling volume for each sample was calculated. DNA libraries were normalized to 4 nM and pooled into a single microtube. After mixing, libraries were prepared using MiSeq ^®^ Reagent Kit V3 600 cycles kit (Illumina^®^), the final pooled libraries were denatured with NaOH 0.2 N, then diluted with hybridization buffer before sequencing, PhiX was used as an internal control for each run. The final pooled library concentration and size were checked using Qbit and Qsep. The counts per reaction for each microbiome species was recorded. PhiX Control v3 (Illumina^®^) was used as an internal control. The pooled libraries were sequenced on an Illumina MiSeq platform following the manufacturer’s standard protocol. Sequencing data from the bacterial 16S rRNA V3–V4 hypervariable region were analyzed using the 16S Metagenomics app. Subsequent sequence processing and downstream analyses were performed using Quantitative Insights into Microbial Ecology 2 (QIIME2; versions 2020.2 and 2020.6) [27]. DNA reads under 200 bp were omitted from the taxonomic analysis. The remaining reads were demultiplexed and denoised using DADA2. Reads were then demultiplexed and denoised with DADA2 [28]. Denoised reads were trimmed at 15 bp and truncated at 260 bp, and chimeric sequences were removed, resulting in 3,994,640 processed reads for downstream analyses. Potential environmental contaminants were identified and removed using the R package decontam (version 1.8.0) before diversity and taxonomic analyses [29].

### Appendix A.2. Appendix Tables

**Table A1:** Comparison of Neonatal outcome among infants over 28 weeks gestation.

	PGF+ [*n* = 150]	PGF− [*n* = 112]	*p*
Gestational age, weeks	34.0 [33.0, 36.0]	37.0 [34.0, 38.0]	<0.001
Birth weight, g	2215.5 [1933.3, 2547.0]	3106.0 [2647.0, 3311.3]	<0.001
Admission hypothermia	119 (79.3%)	75 (67.0%)	0.032
Respiratory distress syndrome	45 (30.0%)	27 (24.1%)	0.329
symptomatic PDA	1 (0.7%)	2 (1.8%)	0.578
NEC or FI	13 (8.7%)	18 (16.1%)	0.082
IVH	92 (61.3%)	57 (50.9%)	0.102
PVL	36 (24.0%)	22 (19.6%)	0.453
Sepsis	3 (2.0%)	2 (1.8%)	>0.999
ROP requiring treatment	0 (0.0%)	0 (0.0%)	>0.999
PN duration	4.0 [3.0, 6.0]	3.0 [0.75, 5.25]	0.027
Time to Full feeding, d (>100 mL/kg/d)	5.0 [4.0, 6.0]	5.0 [4.0, 6.0]	0.248
Length of stay	16.0 [10.0, 25.0]	12.0 [8.0, 28.0]	0.075
Mortality	1 (0.7%)	0 (0.0%)	>0.999

Values are presented with median [IQR] for continuous variables or frequencies with percentages for categorical variables, as appropriate. *p*-values were calculated using the chi-square test or Fisher’s exact test for categorical variables (as expected cell counts were all greater than 5, Pearson’s chi-square test was initially considered; however, to ensure exact probabilities, Fisher’s exact test was employed.) and the Mann–Whitney U test for continuous variables. Abbreviations: FI, feeding intolerance; IVH, intraventricular hemorrhage; NEC, necrotizing enterocolitis; PDA, patent ductus arteriosus; PGF, postnatal growth failure; PN, parenteral nutrition; PVL, periventricular leukomalacia; ROP, retinopathy of prematurity.

**Table A2:** The average Relative abundance in Phylum level, at meconium of neonates.

Phylum	Overall Mean (%)	PGF+ [*n* = 151]	PGF− [*n* = 131]	*p*
Firmicutes	32.864020	30.641	35.427	0.049773
Proteobacteria	32.583643	36.597	27.957	0.00543
Bacteroidetes	27.473766	26.186	28.958	0.251026
Actinobacteria	4.578797	4.404	4.781	0.537637
Tenericutes	1.284061	0.995	1.618	0.380373
Others	1.215713	1.178	1.259	0.969634

Values are presented as mean relative abundance (%). *p* values were calculated using the Mann–Whitney U test. Others indicate the sum of all remaining phyla excluding the top 5 phyla listed above. *Abbreviations*: PGF, postnatal growth failure.

**Table A3:** The average Relative abundance in Genus level, at meconium of neonates.

Genus	Overall Mean (%)	PGF+ [*n* = 151]	PGF− [*n* = 131]	*p*
*Bacteroides*	11.463	11.336	11.610	0.980143
*Prevotella*	10.015	9.219	10.932	0.064222
*Streptococcus*	8.865	9.025	8.682	0.382876
*Ralstonia*	8.673	10.688	6.350	0.010959
*Acidovorax*	4.623	5.563	3.540	0.123708
*Bifidobacterium*	3.533	3.664	3.382	0.504368
*Sutterella*	3.093	2.942	3.268	0.257714
*Staphylococcus*	2.853	1.968	3.873	0.354757
*Sphingomonas*	2.532	2.635	2.414	0.197856
*Megamonas*	2.273	2.074	2.502	0.032141
*Pelomonas*	2.055	2.906	1.074	0.003749
*Veillonella*	2.035	2.078	1.984	0.565024
*Faecalibacterium*	1.896	1.859	1.938	0.277964
*Alloprevotella*	1.624	1.476	1.795	0.110213
*Dialister*	1.573	1.455	1.709	0.080183
*Roseburia*	1.570	1.445	1.714	0.111498
*Enterococcus*	1.475	0.763	2.294	0.157695
*Escherichia*/*Shigella*	1.456	0.990	1.993	0.378898
*Barnesiella*	1.407	1.231	1.609	0.043994
*Brevundimonas*	1.355	1.171	1.567	0.248952
Others	25.632271	25.514028	25.768565	0.967300

Values are presented as mean relative abundance (%). *p* values were calculated using the Mann–Whitney U test. Others indicate the sum of all remaining genera excluding the top 20 genera listed above. *Abbreviations*: PGF, postnatal growth failure.

**Table A4:** Spearman correlation coefficient with GA and the relative abundance.

	PGF+ [*n* = 151]					PGF− [*n* = 131]				
	Spearman’s *ρ*	95% CI Lower	95% CI Upper	Raw *p*	*q*	Spearman’s *ρ*	95% CI Lower	95% CI Upper	Raw *p*	*q*
Firmicutes	0.053	−0.1074	0.2112	0.516	0.860	0.151	−0.0208	0.3147	0.085	0.141
Proteobacteria	−0.102	−0.2578	0.0584	0.212	0.860	−0.066	−0.2349	0.1067	0.453	0.567
Bacteroidetes	0.065	−0.0956	0.2225	0.427	0.860	0.174	0.0026	0.3355	0.047	0.132
Actinobacteria	−0.014	−0.1731	0.1463	0.867	0.941	0.170	−0.0019	0.3315	0.053	0.132
Tenericutes	0.006	−0.1538	0.1656	0.941	0.941	−0.048	−0.2177	0.1246	0.586	0.586
*Bacteroides*	0.029	−0.1315	0.1877	0.725	0.806	0.186	0.0151	0.3466	0.033	0.067
*Prevotella*	0.095	−0.0653	0.2514	0.244	0.563	0.166	−0.0061	0.3278	0.059	0.107
*Streptococcus*	−0.029	−0.1880	0.1312	0.723	0.806	0.106	−0.0663	0.2730	0.226	0.323
*Ralstonia*	−0.078	−0.2353	0.0823	0.338	0.578	−0.070	−0.2390	0.1024	0.425	0.531
*Acidovorax*	−0.099	−0.2542	0.0622	0.229	0.563	0.000	−0.1716	0.1714	0.999	0.999
*Bifidobacterium*	0.009	−0.1511	0.1683	0.915	0.963	0.189	0.0180	0.3492	0.031	0.067
*Sutterella*	0.100	−0.0603	0.2560	0.220	0.563	0.215	0.0452	0.3728	0.014	0.047
*Staphylococcus*	−0.122	−0.2764	0.0384	0.135	0.563	−0.049	−0.2182	0.1240	0.582	0.685
*Sphingomonas*	−0.182	−0.3322	−0.0231	0.025	0.504	−0.147	−0.3109	0.0249	0.093	0.144
*Megamonas*	0.094	−0.0670	0.2497	0.252	0.563	0.214	0.0439	0.3717	0.014	0.047
*Pelomonas*	−0.068	−0.2249	0.0932	0.410	0.585	−0.096	−0.2630	0.0770	0.276	0.368
*Veillonella*	−0.002	−0.1613	0.1581	0.984	0.984	0.190	0.0194	0.3503	0.030	0.067
*Faecalibacterium*	0.117	−0.0438	0.2714	0.153	0.563	0.303	0.1392	0.4515	0.000	0.009
*Alloprevotella*	0.094	−0.0672	0.2496	0.253	0.563	0.203	0.0327	0.3620	0.020	0.057
*Dialister*	0.087	−0.0739	0.2432	0.289	0.578	0.220	0.0507	0.3776	0.011	0.047
*Roseburia*	0.102	−0.0589	0.2574	0.213	0.563	0.222	0.0522	0.3788	0.011	0.047
*Enterococcus*	−0.039	−0.1974	0.1216	0.636	0.806	−0.015	−0.1858	0.1572	0.867	0.964
*Escherichia*/*Shigella*	0.034	−0.1266	0.1925	0.680	0.806	0.223	0.0539	0.3803	0.010	0.047
*Barnesiella*	0.072	−0.0889	0.2289	0.381	0.585	0.160	−0.0117	0.3228	0.068	0.113
*Brevundimonas*	−0.077	−0.2340	0.0836	0.347	0.578	−0.002	−0.1738	0.1693	0.979	0.999

Spearman’s rank correlation analyses were performed to evaluate the association between gestational age and relative abundance of selected microbial taxa in the PGF+ and PGF− groups. Values are presented as Spearman’s correlation coefficient (*ρ*), 95% confidence interval (CI), raw *p* value, and Benjamini–Hochberg false discovery rate-adjusted *q* value. FDR correction was applied separately within each PGF group and taxonomic level. Taxa with *q* < 0.05 were considered statistically significant after correction. Abbreviations: CI, confidence interval; FDR, false discovery rate; GA, gestational age; PGF, postnatal growth failure.

**Table A5:** Adjusted distance-based redundancy analysis of meconium microbiome composition.

Distance Metric	Model/Effect	Adjustment Variables	F	R^2^	P
Bray–Curtis	Overall adjusted model	PGF status, GA, birth weight	2.271	0.0239	0.014
Bray–Curtis	PGF effect adjusted for GA and birth weight	GA, birth weight	2.367	0.0083	0.047
Jaccard	Overall adjusted model	PGF status, GA, birth weight	1.257	0.0134	0.047
Jaccard	PGF effect adjusted for GA and birth weight	GA, birth weight	1.218	0.0043	0.156

The overall model tested the explanatory contribution of the full model including PGF status, gestational age, and birth weight. The PGF effect tested the independent association of PGF status after adjustment for gestational age and birth weight. Statistical significance was assessed using permutation-based tests with 999 permutations. For the overall models, *df* = 3 and residual *df* = 278; for the adjusted PGF effects, *df* = 1 and residual *df* = 278. Abbreviations: GA, gestational age; PGF, postnatal growth failure.
